# 2-Nitrophenol sensor-based wet-chemically prepared binary doped Co_3_O_4_/Al_2_O_3_ nanosheets by an electrochemical approach†

**DOI:** 10.1039/c7ra10866d

**Published:** 2018-01-03

**Authors:** Mohammed M. Rahman, M. M. Alam, Abdullah M. Asiri

**Affiliations:** Chemistry Department, King Abdulaziz University, Faculty of Science P.O. Box 80203 Jeddah 21589 Saudi Arabia; Center of Excellence for Advanced Material Research (CEAMR), King Abdulaziz University P.O. Box 80203 Jeddah 21589 Saudi Arabia; Department of Chemical Engineering and Polymer Science, Shahjalal University of Science and Technology Sylhet 3100 Bangladesh mmalamsust@gmail.com +880-821548976

## Abstract

Herein, the wet-chemical process (co-precipitation) was used to prepare nanosheets (NSs) of Co_3_O_4_/Al_2_O_3_ in an alkaline medium (pH ∼ 10.5). The synthesized NSs were totally characterized by Fourier-transform infrared spectroscopy (FTIR), ultraviolet visible spectroscopy (UV/vis), field emission scanning electron microscopy (FESEM), energy-dispersive X-ray spectroscopy (EDS), X-ray photoelectron spectroscopy (XPS), and powder X-ray diffraction (XRD). The synthesized NSs were deposited onto a glassy carbon electrode (GCE) to prepare a very thin layer with a conducting binder for detecting 2-nitrophenol (2-NP) selectively by a reliable electrochemical method. The proposed chemical sensor exhibits good sensitivity (54.9842 μA μM^−1^ cm^−2^), long-term stability, and enhanced chemical response by electrochemical approaches. The resultant current is found to be linear over the concentration range (LDR) from 0.01 nM to 0.01 mM. The estimated detection limit (DL) is equal to 1.73 ± 0.02 pM. This study introduces a potential route for future sensitive sensor development with Co_3_O_4_/Al_2_O_3_ NSs by an electrochemical approach for the selective detection of hazardous and carcinogenic chemicals in environmental and health care fields.

## Abstract 2

This potential research work introduces a route of future sensitive sensor development with Co_3_O_4_/Al_2_O_3_ NSs by electrochemical approach to selective detection of hazardous and carcinogenic chemicals in environmental and health care fields.

## Graphical abstract

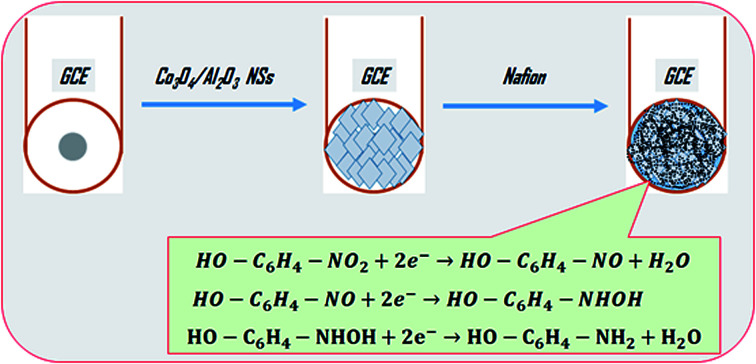

## 1. Introduction

Nitro-organic compounds (nitro-phenols) are well known as anthropogenic, toxic, inhibitory, and bio-refractory compounds and have vast industrial applications in the manufacture of a variety of useful products such as pharmaceuticals, industrial chemicals, pesticides, organic dyes, fungicides, insecticides, explosives, and aniline.^1^ Therefore, the EPA (Environmental Protection Agency) and EU (European Union) have enlisted NPs as hazardous because of their highly toxic behavior towards the environment including human, animal, plants, aquatic life, and living organisms.^2–4^ NPs may cause various harmful effects in human body, particularly cancer, generate poisoning, and tumors in the urinary tract.^5,6^ The NPs are soluble in water, and due to the numerous manmade activities, they have not only been detected in industrial effluents, but also in fresh water and marine environment.^7^ To protect the public health and the environment from harmful effects of NPs, it is obligatory to develop an efficient and sensitive portable device (chemical sensor) that will be used to detect NPs in the working place, environment, and public health sector.^8^ The existing typical methods, such as spectrophotometry,^9^ fluorescence,^10^ gas chromatography,^11^ capillary electrophoresis,^12^ and high performance liquid chromatography,^13^ are applied to detect NPs. However, these traditional methods have several disadvantages such as being costly and time consuming, requiring heavy instrumentation, and are complicated to use outdoors. On the other hand, the electrochemical method has useful features including low cost, easy operation, short response time, portable simple instrumentation, and selectivity with high sensitivity.^14^

The transition metal oxides with multiple oxidation states such as Fe_2_O_3_, Co_3_O_4_, MnO_2_, and CrO_2_ are extensively applied as a successive electron mediators to detect NPs.^15^ An efficient NP chemical sensor fabricated with α-MnO_2_ nanotubes had exhibited the sensitivity of 19.18 μA mM^−1^ cm^−2^ and detection limit of 0.1 mM.^16^ Another NP chemical sensor based on Mn_2_O_3_/ZnO nanoparticles showed the sensitivity 4.6667 μA μM^−1^ cm^−2^ with the detection limit 0.83 nM.^17^ In this decade, carbon nanotubes impregnated with transition metal oxides are extensively considered for the detection of environmental toxins, and this is becoming increasingly popular ever since the first invention.^18–20^ It has been reported that the NP sensor fabricated with hydride components of carbon nanotube and phthalocyanine cobalt(ii) shows an outstanding detection limit 0.2 μM with a linear dynamic range from 1 μM to 1.9 mM.^21^ Besides this, another talented NP chemical sensor assembled with a poly(diallyldimethylammonium chloride)-functionalized graphene composite has exhibited a dynamic result of detection limit 0.02 μM with a wider linear dynamic range from 0.06 to 110 μM.^22^

Due to the high toxicity of 2-NP, sustainable development of methods for its selective detection is urgently needed. Therefore, an initiative has been taken for efficient detection of 2-NP by an electrochemical sensor constructed with Co_3_O_4_/Al_2_O_3_ NSs. A thin layer of Co_3_O_4_/Al_2_O_3_ NSs with a conducting binding agent (5% Nafion suspension in ethanol) was deposited on GCE to obtain a working electrode of 2-NP chemical sensor. The assembled Co_3_O_4_/Al_2_O_3_ NS/binder/GCE was applied successfully to detect 2-NP by a reliable electrochemical approach. To the best of our knowledge, the exploration of environmental toxin (2-NP) using a chemical sensor fabricated with active Co_3_O_4_/Al_2_O_3_ NSs has been reported for the first time herein. Therefore, it may be concluded that the 2-NP chemical sensor based on Co_3_O_4_/Al_2_O_3_ NSs onto GCE is a novel introduction in the field of sensor technology with promising applications in environmental toxin analysis.

## 2. Experimental

### 2.1 Materials and methods

The analytical grade chemicals cobalt(ii) chloride (CoCl_2_), aluminum chloride (AlCl_3_), and ammonium hydroxide (NH_4_OH) were obtained from the Sigma-Aldrich company and used as received to prepare Co_3_O_4_/Al_2_O_3_ NSs. To execute this study, M-tolylhydrazine (M-THyd), 2,4-dinitrophenol (2,4-DNP), 2-nitrophenol (2-NP), methanol (M), 3-methylaniline (3-MA), 3-chlorophenol (3-CP), ammonium hydroxide (AH), 3,4-diaminotoluene (3,4-DAT), 3-methoxyphenylhydrazine (3-MPHyd), bisphenol A (BPA), Nafion (5% Nafion suspension in ethanol), monosodium phosphate, and disodium phosphate were purchased from Sigma-Aldrich company. To explore the FTIR and UV-vis spectra, the produced Co_3_O_4_/Al_2_O_3_ NSs were analyzed using a Thermoscientific NICOLET iS50 FTIR (Madison, WI, USA) and 300 UV/visible spectrophotometer (Thermoscientific), respectively. The binding energies of Co, Al, and O and the corresponding oxidation states were measured by XPS analysis using a K-α1 spectrometer (thermo scientific, K-α1 1066) with a radiation source (A1Kα1, Beam spot size = 300.0 μm, pass energy = 200.0 eV, and pressure ∼ 10^−8^ torr). The optical properties, such as the arrangement of molecules, analysis of elements, morphological structure, and particle size, of synthesized NSs were investigated by implementation of FESEM (JEOL, JSM-7600F, Japan) and XEDS. Besides this, the phase (crystallinity of nanoparticles) identification was carried out by the execution of XRD analysis on the prepared Co_3_O_4_/Al_2_O_3_ NSs under ambient conditions. A slurry of NSs was used to coat the GCE with a conducting binder, and the resulting working electrode was implemented successfully to detect 2-NP in an aqueous medium. The electrochemical measurement was executed by a Keithley electrometer (6517A, USA) under room condition.

### 2.2 Preparation of Co_3_O_4_/Al_2_O_3_ NSs by a wet-chemical process

The wet-chemical process (co-precipitation) is an extensively used technique to prepare doped nanomaterials at low temperatures. This method involves three successive steps: (i) precipitation of two or more metal hydroxides in aqueous media, (ii) drying of separated precipitate, and (iii) calcination of dried precipitate in a high temperature muffle furnace. For the execution of this study, the predetermined weights of cobalt chloride (CoCl_2_) and aluminum chloride (AlCl_3_) were dissolved in 100.0 mL de-ionized (DI) water in a 250.0 mL conical flask. Then, a 0.1 M solution of NH_4_OH was added to the resultant solution dropwise under continuous magnetic stirring, and the pH of the solution was adjusted to around 10.5. Under this condition, the metal ions were precipitated out quantitatively in the form of Co(OH)_2_/Al(OH)_3_. The precipitate was separated out from water and then dried in an oven at 105 °C. Subsequently the dried sample was calcined at 500 °C for 6 hours in a high temperature furnace for the metal hydroxides to transform into the metal oxide form Co_3_O_4_/Al_2_O_3_ in presence of atmospheric oxygen. The calcined sample in the oxide form was ground in a mortar into nanosized particles. The reaction scheme is supposed to be as follows:

In aqueous medium:

i NH_4_OH_(s)_ → NH_4_^+^_(aq)_ + OH^−^_(aq)_

ii CoCl_2(s)_ → Co^2+^_(aq)_ + 2Cl^−^_(aq)_

iii AlCl_3(s)_ → Al^3+^_(aq)_ + 3Cl^−^_(aq)_

iv Co^2+^_(aq)_ + Al^3+^_(aq)_ + 5OH^−^_(aq)_ + *n*H_2_O → Co(OH)_2_/Al(OH)_3(s)_·*n*H_2_O↓

In the muffle furnace:

v Co(OH)_2_/Al(OH)_3(s)_ + O_2_ → Co_3_O_4_/Al_2_O_3_ + H_2_O_(v)_

**Scheme 1:**
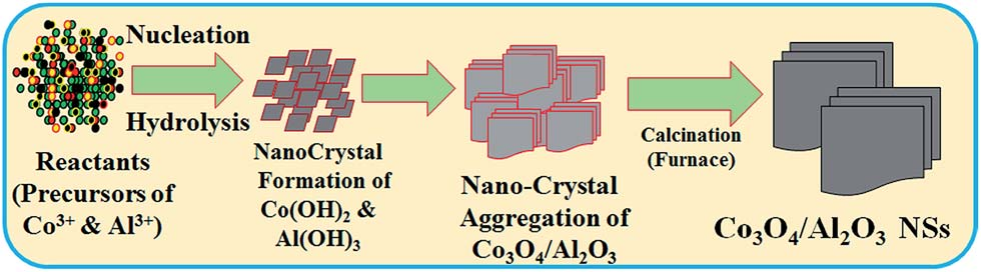
The growth mechanism of Co_3_O_4_/Al_2_O_3_ NSs produced by the low-temperature wet-chemical method.

In the wet-chemical process, the precipitation of metal ion in the form of metal hydroxide is dependent on the value of *K*_s_ (solubility product constant) in an aqueous medium. At the pH 10.5, the *K*_s_ values for Al(OH)_3_ and Co(OH)_2_ are 3 × 10^−34^ and 5.92 × 10^−15^, respectively.^34^ As 0.1 M ammonium hydroxide solution is added dropwise to the resultant solution, the OH^−^ concentration increases gradually. Consequently, with the lower *K*_s_ value of Al(OH)_3_, aluminum hydroxide starts to precipitate first and forms nuclei of crystals.^23^ Then, with an increase in the OH^−^ ion concentration, the crystallites of aluminum hydroxide start to aggregate. Due to the addition of ammonium hydroxide in the resulting solution, the pH continues to increase for the adjustment of the reactor during preparation of nanomaterials. Then, cobalt hydroxide also starts to precipitate, which is adsorbed on crystallites of aluminum hydroxide. This similar tendency of growth pattern of nanomaterials has been reported elsewhere.^24–26^ Subsequently, the obtained crystals of metal hydroxides are sequentially washed with water, ethanol, and acetone and dried overnight at 105 °C in an oven. After this, the dried sample is subjected to calcination at higher temperatures in a furnace (Barnstead Thermolyne, 6000 Furnace, USA) at 500 °C for 6 hours. The schematic of the formation mechanism of Co_3_O_4_/Al_2_O_3_ NSs is presented in Scheme 1. To obtain the nano-sized particles, the calcined Co_3_O_4_/Al_2_O_3_ NSs were ground in a mortar. The prepared NSs were applied for the detection of 2-NP by an electrochemical approach under ambient conditions.

### 2.3 Fabrication of GCE with Co_3_O_4_/Al_2_O_3_ NSs

A slurry of Co_3_O_4_/Al_2_O_3_ NSs was prepared in ethanol and coated on a GCE as a very thin layer. To enhance the physical binding strength between NSs and GCE, a drop of Nafion (5% Nafion suspension in ethanol) was added. The assembled Co_3_O_4_/Al_2_O_3_ NS/binder/GCE was kept inside an oven at 34.0 °C for enough time to dry the conducting film entirely. The desired electrochemical cell was organized with the Co_3_O_4_/Al_2_O_3_NS/binder/GCE and a Pt-wire (diameter 1.5 mm) as the working electrode and the counter electrode, respectively. To execute the sensor analytical performances, a series of 2-NP solutions based on the concentration ranging from 0.01 nM to 0.1 mM was prepared and used as a target analyte. The sensitivity of the anticipated chemical sensor was estimated from the slope of the calibration curve accomplished as resultant-current *versus* applied-concentration of 2-NP. The linear dynamic range (LDR) was measured from the maximum linearity (regression coefficient, *r*^2^) of calibration curve, and detection limit was obtained from the signal to noise ratio of 3. The used electrometer is a simple two-electrodes system. The volume of the buffer solution (PBS) in the measuring electrochemical cell was maintained constant at 10.0 mL during the execution of this study.

## 3. Results and discussions

### 3.1 Structural analyses

**Fig. 1:**
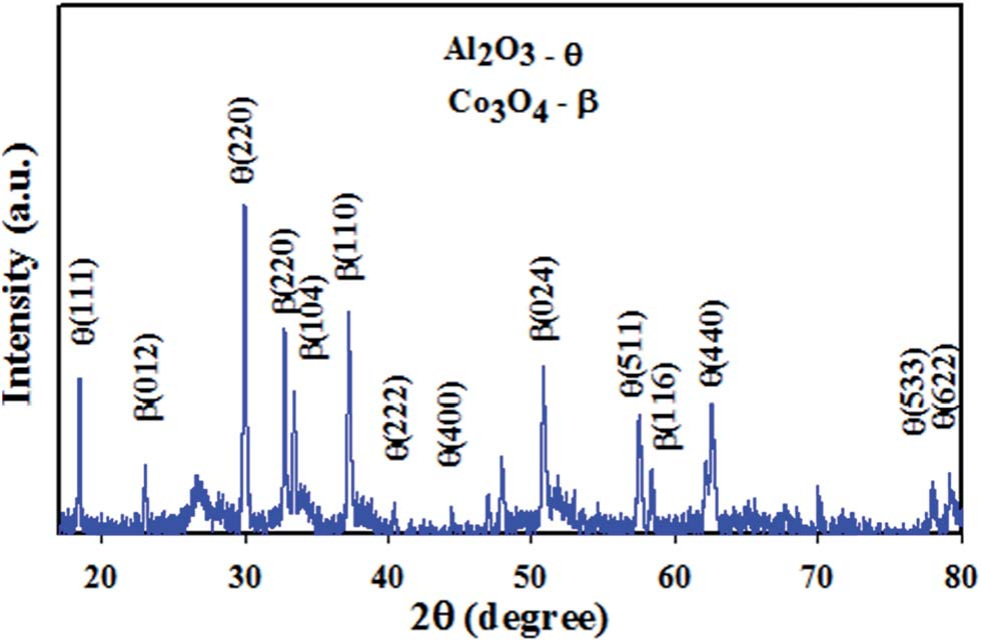
Powder XRD pattern of the Co_3_O_4_/Al_2_O_3_ nanostructures for the structural analysis.

The XRD (powder X-ray diffraction) patterns are able to provide detailed information about unit cell dimensions (phase crystallinity), and this technique was implemented on Co_3_O_4_/Al_2_O_3_ NSs with the radiation source Cu–Kα1 (*λ* = 1.54178 Å) in the range of 10–80°, and the corresponding scanning speed was 2° min^−1^. According to the XRD spectrum presented in Fig. 1, the synthesized Co_3_O_4_/Al_2_O_3_ NSs contain well-assorted phases of Co_3_O_4_ and Al_2_O_3_. As observed from the Fig. 1, the reflected peaks of Co_3_O_4_ indices as *β* are (111), (220), (222), (400), (533), (622), (440), and (511), having great similarities with the previous reports.^27–30^ Beside this, the observed diffracted peaks for Al_2_O_3_ indices as *θ* are (012), (104), (024), (220), (110), and (116), which have been identified by previous authors^31–35^ and JCPDS no. 29-0063 to be belonging to Al_2_O_3_. Based on the Scherrer equation, the crystal size of the nanoparticles can be calculated from the XRD diffraction pattern.

vi *D* = 0.9l*λ*/(*β* cos *θ*)

Herein, *λ* represents the wavelength (X-ray radiation = 1.5418 Å) and *β* is full width at half maxima (FWHM), corresponding to most intense peak, and *θ* is the diffracting angle.^36^ From the eqn (vi), the calculated crystal size (following the Scherrer equation) is equal to 30.86 nm. The optical analysis of Co_3_O_4_/Al_2_O_3_ NSs is also performed and presented in the ESI section (Fig. S4†).

### 3.2 Morphological and elemental analyses

**Fig. 2:**
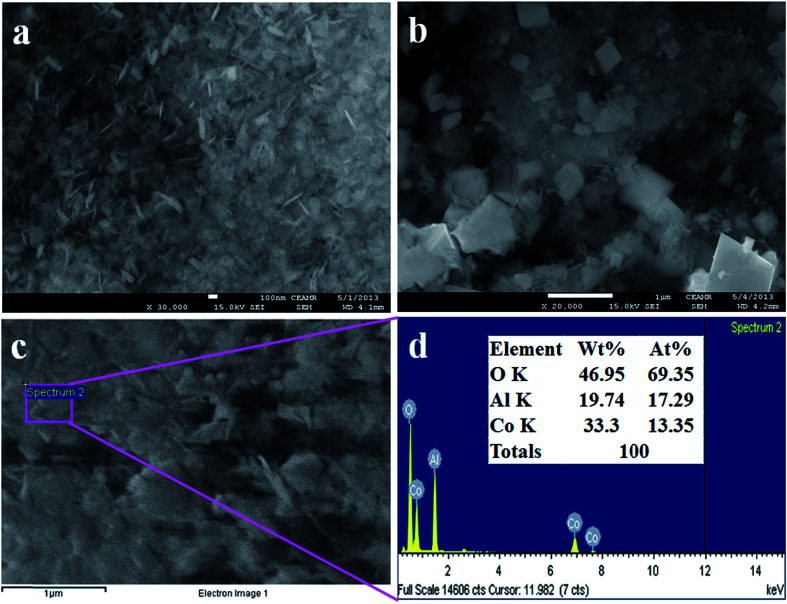
Morphological and elemental analyses (a and b) FESEM analysis from high to low magnified images and (c and d) elemental analysis of Co_3_O_4_/Al_2_O_3_ nanostructures.

The morphological and elemental analysis of synthesized Co_3_O_4_/Al_2_O_3_ NSs were investigated by FESEM and EDS analysis, as illustrated in Fig. 2. The typical FESEM images with high to low magnification are presented in the Fig. 2(a and b), and it is clearly visible that the synthesized Co_3_O_4_/Al_2_O_3_ materials are nanosheets in terms of shape.^37–39^ The FESEM investigation is similar with the results of EDS analysis, as shown in Fig. 2(c and d). According to the EDS analysis, the composition of Co_3_O_4_/Al_2_O_3_ NSs is O 46.95%, Al 19.74%, and Co 33.3%. Besides these, any other peak is not visible; therefore, it can be concluded that the prepared Co_3_O_4_/Al_2_O_3_ NSs consist of cobalt, aluminum, and oxygen only.^40–42^

### 3.3 Binding energy analysis

**Fig. 3:**
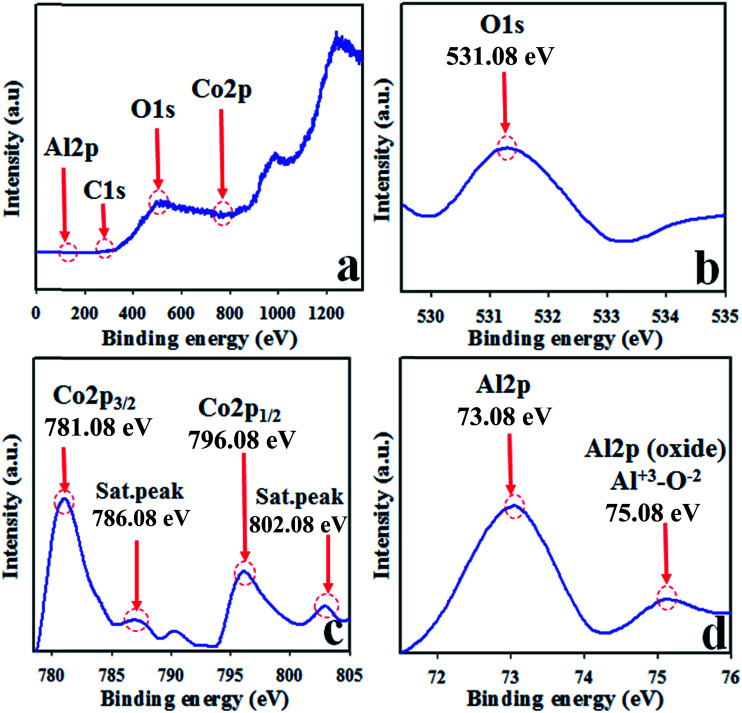
Binding energy analysis by XPS study of Co_3_O_4_/Al_2_O_3_ NSs (a) full spectrum, (b) O 1s level, (c) spin orbit Co 2p level, and (d) spin orbit of Al 2p level.

The synthesized NSs were subjected to XPS investigation with an X-ray beam. The XPS spectrum is obtained when the atoms of the sample absorb the kinetic energy of X-ray beam and the electrons of outer spin orbital jump from lower to higher energy level. The atomic composition with the corresponding chemical formula and the oxidation state of the species present in the nanomaterial can be scrutinized efficiently by this practice.^43–45^ The core level XPS spectra of Co 2p, Al 2p, and O 1s are presented in Fig. 3. The high resolution of Co 2p spectrum shows two obvious dominated peaks centered at 781.0 and 796.0 eV. These two sharp peaks can be ascribed to Co 2p_3/2_ and Co 2p_1/2_ orbits and obviously verify the presence of Co^3+^. The projected two satellite peaks of Co 2p_3/2_ and Co 2p_1/2_ are at 787.0 and 803.0 eV, respectively. These two peaks can be attributed to the presence of Co^2+^ in the synthesized NSs, as illustrated in Fig. 3(c).^46–50^ Therefore, the presence of dominant and satellite peaks of Co 2p spin orbitals indicate the co-existence of Co(ii) and Co(iii) on the surface of synthesized Co_3_O_4_/Al_2_O_3_ NSs. The O 1s exhibit a peak at 531.3 eV, which is presented in Fig. 3(b) and attributed to O^2−^ in Al_2_O_3_.^51–55^ The XPS spectra of Al 2p is concentrated at 73.3 and 75.1 eV, and the peaks of Al 2p at 75.1 eV are ascribed to Al^3+^–O^2−^ bonds in Al_2_O_3_, as illustrated in Fig. 3(d).^56–59^ The different states of O 1s, Al 2p, and Co 2p are also quantified and presented in the ESI section (Fig. S5†).

### 3.4 Applications: sensing of 2-NP by Co_3_O_4_/Al_2_O_3_ NSs

**Scheme 2:**
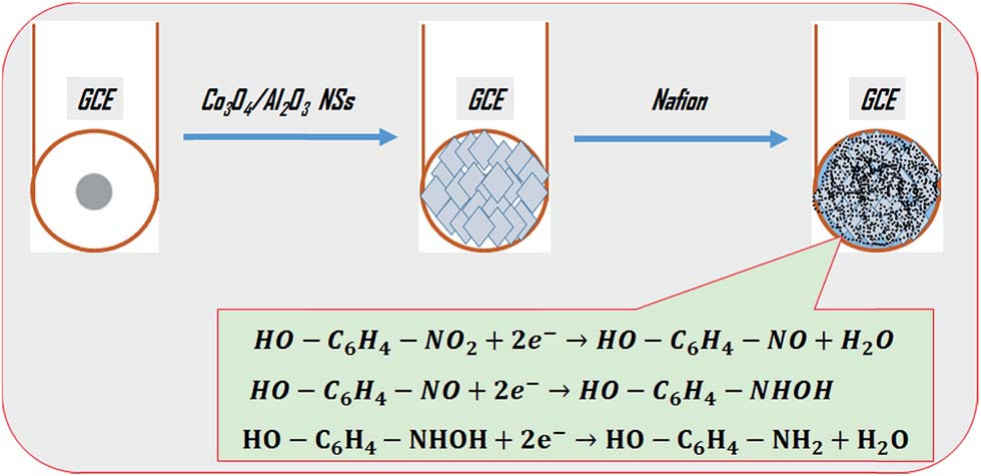
The expected sensing mechanism for the determination of 2-NP by Co_3_O_4_/Al_2_O_3_ NSs on sensor probe.

The selective detection of 2-NP was executed in the optimized buffer system, and this performance was carried out by the implementation of Co_3_O_4_/Al_2_O_3_ NS/binder/GCE as the working electrode. To enhance the binding strength between NSs and GCE, a drop of Nafion was added. Nafion not only enhances the binding strength, but also increases stability, conductivity, and electron transfer rate of the electrode.^60,61^ Thus, the prepared electrode exhibited the advantages of high stability in air as well as in chemical environment, enhanced electrochemical performance during the detection of target analyte (2-NP), and can be easily subjected to performances, assembling, and fabrication, and above all, safe chemo-characteristics. Therefore, the proposed chemical sensor Co_3_O_4_/Al_2_O_3_ NS/binder/GCE was successfully used to detect 2-NP in an aqueous medium. During the performance of 2-NP chemical sensor, applied *I*–*V* was measured on thin-film of Co_3_O_4_/Al_2_O_3_ NS/binder/GCE, and the holding time in the electrometer was set as 1.0 s. A possible reduction mechanism of 2-NP is illustrated in Scheme 2. As observed from Scheme 2, the electrons are accepted from the applied current to reduce 2-NP into 2-AP (2-aminophenol). Therefore, the species of reactive 2-NP are adsorbed on the Co_3_O_4_/Al_2_O_3_ NS surface and reduced to 2-aminophenol, as shown in reaction (vii) to (ix). Since the electrons are required to reduce 2-NP, the electrochemical response (*I*–*V*) is inversely proportional to the corresponding concentration of 2-NP.^62–65^ A schematic of the detection process of 2-NP based on Co_3_O_4_/Al_2_O_3_ NS/binder/GCE sensor is demonstrated in Scheme 2.

The possible suggested reduction mechanism of 2-NP is presented in the reaction (vii) and (ix).

vii HO − C_6_H_4_ − NO_2_ + 2e^−^ → HO − C_6_H_4_ − NO + H_2_O

viii HO − C_6_H_4_ − NO + 2e^−^ → HO − C_6_H_4_ − NHOH

ix HO − C_6_H_4_ − NHOH + 2e^−^ → HO − C_6_H_4_ − NH_2_ + H_2_O

**Fig. 4:**
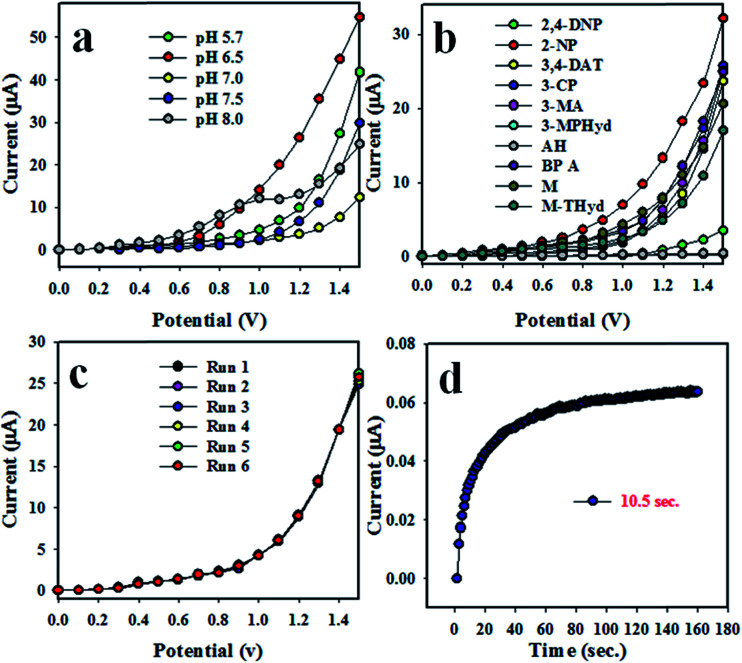
Optimization of 2-NP with Co_3_O_4_/Al_2_O_3_ NSs. (a) pH optimization, (b) selectivity, (c) repeatability, and (d) response time.

The synthesized NSs are not responsive equally in all buffers to applied *I*–*V*. Therefore, for obtaining the maximum output of the *I*–*V* responses, the pH of the measuring buffer system was necessary to be optimized for the Co_3_O_4_/Al_2_O_3_ NS/binder/GCE. Fig. 4(a) represents the *I*–*V* response of pH ranging from 5.7 to 8.0. Obviously, among the tested buffer system, the best *I*–*V* response was attained at pH 6.5. Then, a number of environmental toxins have been investigated at the concentration level of 1.0 μM and pH = 7.5 with the proposed chemical sensor. The electrochemical responses of M-THyd, 2,4-DNP, 2-NP, methanol, 3-MA, 3-CP, AH, 3,4-DAT, 3-MPHyd, and BPA are illustrated in Fig. 4(b). Obviously, the electrochemical response of 2-NP has the highest intensity under experimental conditions. The reproducibility test of a chemical sensor provides the evidence of reliability. Therefore, this test was performed at 0.1 μM concentration of 2-NP, and the resulting data is represented in Fig. 4(c). As seen in the Fig. 4(c), the replicated six runs are practically indistinguishable under an identical condition. The electrochemical responses are unchanged even after washing of electrode in each trial. Therefore, this test provides the evidence of reliability, and the projected 2-NP chemical sensor can be applied in the real field successively. The relative standard deviation of the reproducibility performances (RSD) is estimated, and it is found to be 1.54 at an applied potential of +1.5 V. The response time is another tool to measure the efficiency of a chemical sensor, and this test has been performed using a 0.1 μM solution of 2-NP. As illustrated in Fig. 4(d), steady response is achieved in about around 10.5 s, and the obtained result may be considered highly satisfactory.

**Fig. 5:**
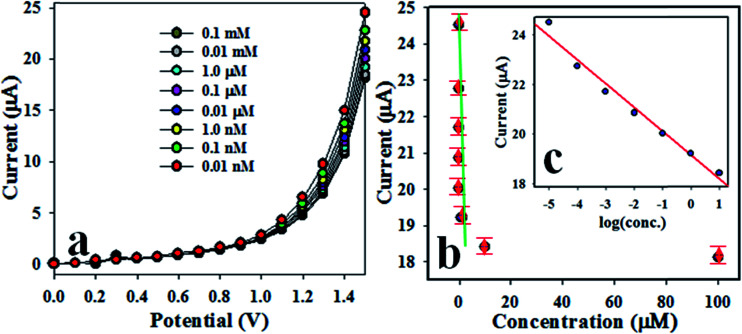
Performance of 2-NP sensor. (a) Concentration variation of 2-NP based on Co_3_O_4_/Al_2_O_3_ NSs/GCE by *I*–*V* method, (b) calibration curve (inset: log[2-NP conc.] *vs.* current).

Fig. 5(a) presents the *I*–*V* response of 2-NP for various concentrations ranging from 0.01 nM to 0.1 mM. Evidently, this is a very wide range, and the applied potential is higher than +1.0 V. As shown in Fig. 5(a), *I*–*V* responses are distinguishable from lower to higher concentration of 2-NP. To evaluate the analytical performance of the projected chemical sensor, the current data at an applied potential of +1.5 V has been obtained from Fig. 5(a) and plotted as current *vs.* concentration of 2-NP in the Fig. 5(b), which is known as a calibration plot. This calibration plot is found to be linear with the regression co-efficient value *r*^2^ = 0.99 along the *x*-axis in logarithmic scale. The sensitivity of 2-NP chemical sensor is estimated from the slope of calibration curve, and it is equal to 54.9842 μA μM^−1^ cm^−2^. The linear dynamic range (LDR) is found to be from 0.01 nM to 0.01 mM. At a signal to noise ratio equal to 3, the limit of detection (LOD) and limit of quantity (LOQ) were calculated to be 1.73 ± 0.02 pM and 5.77 ± 0.02 pM, respectively. Obviously, the Co_3_O_4_/Al_2_O_3_ NS/binder/GCE electrode could be used to determine 2-NP in an aqueous medium in a wide concentration range. The stability performance of electrode based on Co_3_O_4_/Al_2_O_3_ NSs has been evaluated in the detection of 2-NP under identical conditions for intra-days and inter-day, as presented in Fig. S1 and S2,† respectively, in the ESI section.† Therefore, it can be concluded from Fig. S1 and S2† that the fabricated electrode is able to perform efficiently in long-term. The reliable measurement of 2-NP with other toxins such as 3-CP and 4-NP in an aqueous medium is illustrated in Fig. S3 in the ESI.† According to this figure, the toxin 3-CP and 4-NP have not shown any remarkable interference effect on the projected chemical sensor during the sensing of 2-NP.

As observed from Fig. 5(a), the electrochemical response of 2-NP chemical sensor based on active Co_3_O_4_/Al_2_O_3_ NS/GCE increases with a decrease in the concentration of 2-NP. Therefore, the highest *I*–*V* response is found at lowest concentrations of 2-NP. During the sensing of 2-NP by Co_3_O_4_/Al_2_O_3_ NS/GCE electrode, a small surface coverage due to the adsorption of few 2-NP molecules on surface of electrode occurs in the initial stage, and corresponding reduction reaction of 2-NP starts progressively. With enrichment of analyte (2-NP), the rate of reaction is increased, and the higher surface coverage is obtained. With further enrichment of 2-NP concentration in the sensing medium, the reduction reaction comes to equilibrium, and the surface coverage approaches its saturation state. With an additional increase in the analyte concentration, a steady state equilibrium *I*–*V* response is observed, and a saturated surface coverage is accomplished. Therefore, it can be summarized that the proposed 2-NP chemical sensor based on the Co_3_O_4_/Al_2_O_3_ NS/GCE assembly can be applied for efficient detection of the targeted toxin (2-NP). As indicated, the proposed 2-NP chemical sensor exhibits reasonable short response time of around 10.5 s, and it should be mentioned that 10.5 s is needed by the 2-NP chemical sensor to attain steady state saturation. Since the proposed 2-NP chemical sensor demonstrates a high sensitivity of 54.9842 μA μM^−1^ cm^−2^, it can be accredited that it has very good adsorption capacity and active catalytic decomposition ability.^66–70^ The interference effect is studied in the presence of different phenolic compounds and their derivatives, such as 2-NP, 2,4-DNP, BPA, and 3-CP, under identical conditions, as presented in Table 1.

**Table 1:** Measurement of interference effect by Co_3_O_4_/Al_2_O_3_ NS/GCE

Toxins	Observed current^a^ (μA) at +1.5 V				Average^b^ (μA)	RSD^c^ (%)
	*R*_1_	*R*_2_	*R*_3_	*R*_4_		
2-NP	26.756	26.298	25.839	26.347	26.310	1.42
2,4-DNP	3.699	3.613	3.527	3.643	3.621	1.98
BPA	22.839	23.137	22.410	21.981	22.592	2.23
3-CP	23.688	22.875	23.525	24.175	23.566	2.28

a Mean of four repeated (*R*) determination (signal to noise ratio 3) with Co_3_O_4_/Al_2_O_3_ NSs/GCE at 0.1 μM concentration.

b The average observed current of the corresponding toxin.

c Relative standard deviation value indicates precision among four repeated determinations.

According to analytical performance, such as selectivity, detection limit, and dynamic linear range, of the chemical sensor, the proposed 2-NP chemical sensor fabricated based on Co_3_O_4_/Al_2_O_3_ NS/GCE showed a reasonably qualified performance as compared to the chemical sensors based on the various transition nanostructured metal oxides, and a comparison is illustrated in Table 2. In brief, the fabricated 2-NP chemical sensor is simple and efficient to detect 2-NP by applying current *versus* potential electro-chemical approaches. The sensing performance, such as detection limit (LD), linear dynamic range (LDR), and sensitivity, of earlier tested 2-NP chemical sensors are associated and summarized in Table 2.^70–75^

**Table 2:** Comparative performances of various nanomaterials or nanocomposites fabricated electrode for the detection of 2-NP by electrochemical approaches^a^

Materials	LOD	LDR	Sensitivity	Ref.
Ag_2_O NPs/AuE	0.19 μM	1.0 μM to 0.5 mM	0.0474 μA μM^−1^ cm^−2^	70
CuO nanohybrides	0.67 nM	1.0 nM to 1.0 mM	0.045 μA μM^−1^ cm^−2^	71
Spinel ZnMn_2_O_4_	20.0 μM	50.0 μM to 0.05 M	1.5 μA μM^−1^ cm^−2^	72
B-doped diamond electrodes	8.4 mM	—	0.3943 μA μM^−1^ cm^−2^	73
Mn_2_O_3_–ZnO NPs/AgE	0.83 nM	100 pM to 50.0 μM	0.6667 μA μM^−1^ cm^−2^	74
Ce_2_O_3_ CNT NCs	60 pM	100 pM to 100 μM	0.0016 μA μM^−1^ cm^−2^	75
Co_3_O_4_/Al_2_O_3_ NSs/GCE	1.73 ± 0.02 pM	0.01 nM to 0.01 mM	54.9842 μA μM^−1^ cm^−2^	This work

a DL (detection limit), LDR (linear dynamic range), nM (nanomole), pM (picomole).

### 3.5 The analysis of a real sample

The proposed 2-NP chemical sensor based on Co_3_O_4_/Al_2_O_3_ NSs was employed for validation to detect and quantify 2-NP in real samples to test its applicability in practical field. The samples were obtained from various sources such as industrial effluent, extracts from PC baby-bottle, and seawater. The results of the analyses are presented in Table 3 and seem quite satisfactory.

**Table 3:** Measured concentration of 2-NP analytes in real environmental samples

Sample	Added 2-NP concentration	Determined 2-NP concentration^a^ by Co_3_O_4_/Al_2_O_3_ NSs/GCE	Recovery^b^ (%)	RSD^c^ (%) (*n* = 3)
Industrial effluent	0.100 μM	0.0991 μM	99.1	2.11
	0.100 μM	0.1032 μM	103.2	
	0.100 μM	0.1022 μM	102.2	
Plastic baby bottle	0.100 μM	0.1019 μM	101.9	0.83
	0.100 μM	0.1032 μM	103.2	
	0.100 μM	0.1035 μM	103.5	
	0.100 μM	0.1053 μM	105.3	
Sea water	0.100 μM	0.1059 μM	105.9	1.02
	0.100 μM	0.1074 μM	107.4	

a Mean of three repeated determinations (signal to noise ratio 3) with Co_3_O_4_/Al_2_O_3_ NSs/GCE.

b Concentration of 2-NP determined/concentration taken.

c Relative standard deviation value indicates precision among three repeated determinations.

## 4. Conclusions

The reliable wet-chemical process was used to prepare NSs of Co_3_O_4_/Al_2_O_3_ at low temperature. Later, a thin layer of NSs was deposited onto GCE with a conducting binder to fabricate the working electrode for the 2-NP chemical sensor development. The calcined NSs were characterized by FESEM, EDS, XPS, FTIR, UV/vis, and XRD and applied to successively detect 2-NP in an aqueous medium. The proposed 2-NP chemical sensor based on Co_3_O_4_/Al_2_O_3_ NSs displayed higher sensitivity, lower detection limit, broad linear dynamic ranges, and selectivity towards 2-NP by reliable *I*–*V* method. The applied current to concentration of 2-NP is linear over the concentration range from 0.01 nM to 0.01 mM with a detection limit of 1.73 ± 0.02 pM, LOQ of 5.77 ± 0.02 pM, and sensitivity of 54.9842 μA μM^−1^ cm^−2^. This approach introduced a well-organized route of efficient chemical sensor development for environmental pollutants in a broad scales.

## Conflicts of interest

There are no conflicts to declare.

## Supplementary Material

RA-008-C7RA10866D-s001
