# Supplementary material for: 2-Nitrophenol sensor-based wet-chemically prepared binary doped Co3O4/Al2O3 nanosheets by an electrochemical approach
Source: RSC Adv. 2018 Jan 3;8(2):960–70. doi: 10.1039/c7ra10866d (PMC9077016; doi:10.1039/c7ra10866d)
Supplement: RA-008-C7RA10866D-s001 [file RA-008-C7RA10866D-s001.pdf]

Electronic Supplementary Material (ESI) for RSC Advances.
This journal is © The Royal Society of Chemistry 2017

2-Nitrophenol sensor based wet-chemically prepared binary doped Co_3O_4/Al_2O_3 nanosheets by electrochemical approach

Mohammed M. Rahman[a,b,*], M.M. Alam[c], Abdullah M. Asiri[a,b]

[a]Chemistry Department, King Abdulaziz University, Faculty of Science, Jeddah 21589, P.O. Box 80203, Saudi Arabia; [b]Center of Excellence for Advanced Material Research (CEAMR), King Abdulaziz University, Jeddah 21589, P.O. Box 80203, Saudi Arabia; [c]Department of Chemical Engineering and Polymer Science, Shahjalal University of Science and Technology, Sylhet 3100, Bangladesh

Supporting information

Optimization of 2-Nitrophenol chemical sensor (Intra-day):

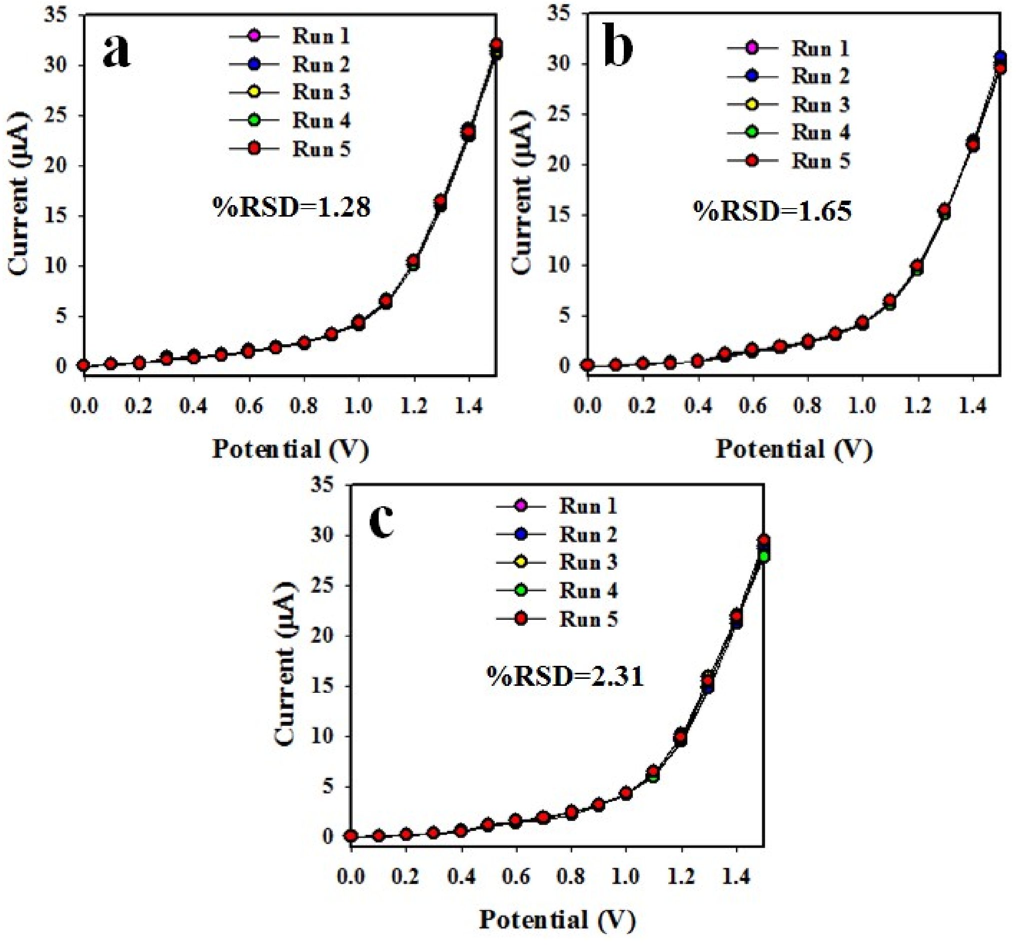

Fig. S1 Optimization of 2-Nitrophenol chemical sensor based on Co_3O_4/Al_2O_3 NSs, (a) repeatability at o hour, (b) repeatability after 2 hour, and (c) repeatability after 4 hours,

Optimization of 2-Nitrophenol chemical sensor (Inter-day):

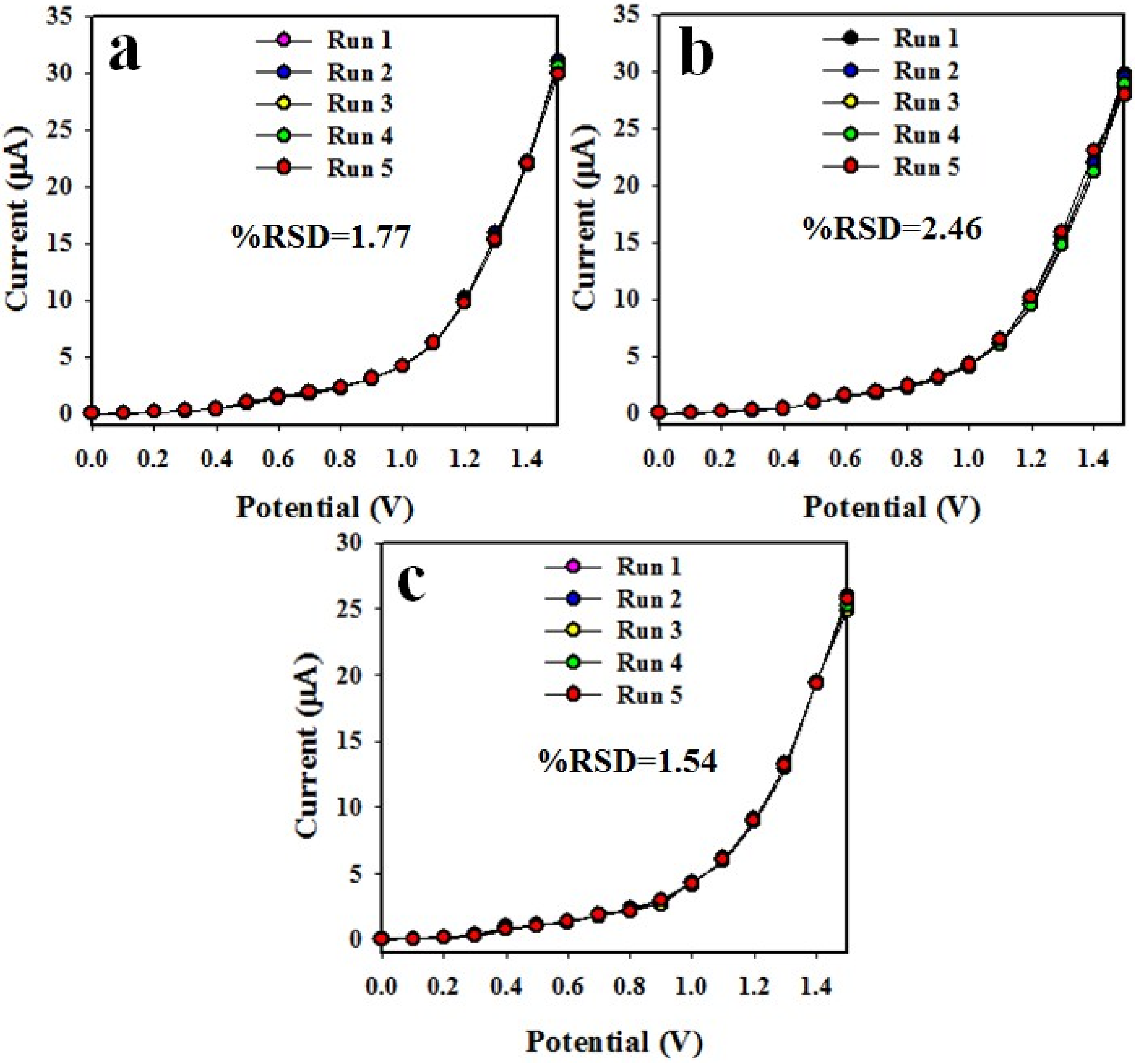

Fig. S2 Optimization of 2-Nitrophenol chemical sensor based on Co_3O_4/Al_2O_3 NSs , (a) repeatability at beginning day, (b) repeatability at 2nd day, and (c) repeatability after 3rd day,

Electrochemical responses in mixtures:

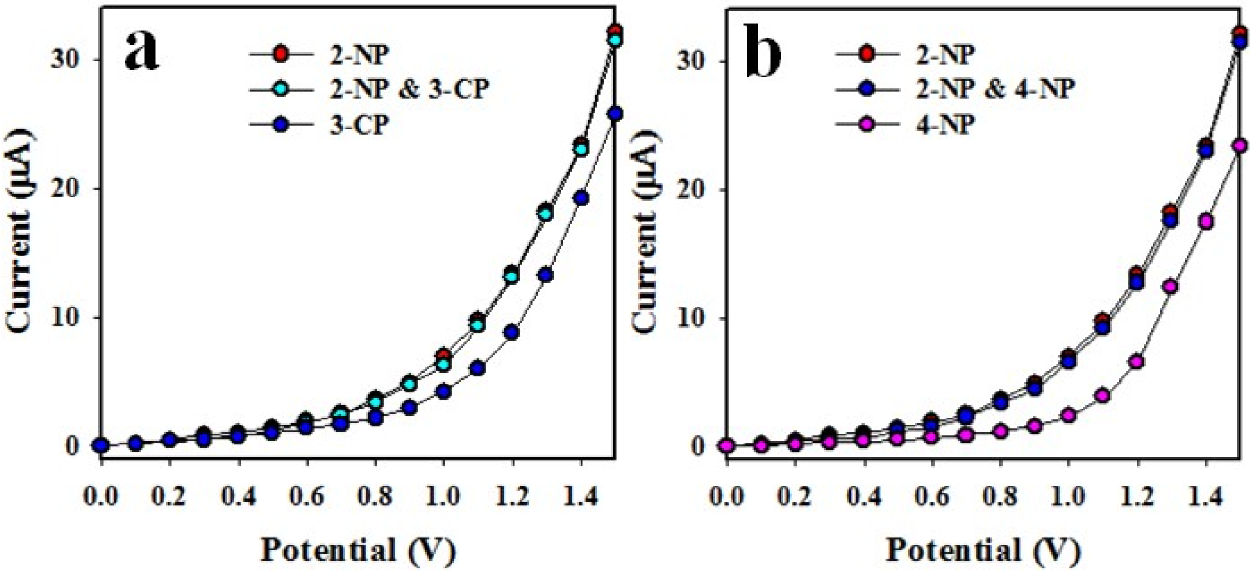

Fig. S3 The reliable control measurements of 2-NP based on Co_3O_4/Al_2O_3 NSs/GCE, (a) I-V responses in mixed or single target chemicals such as 2-NP, 2-NP+3-CP and 3-CP, (b) I-V responses in mixed or single target chemicals such as 2-NP, 2-NP+4-NP and 4-NP.

Optical evaluations:

The photosensitivity of prepared NSs of Co_3O_4/Al_2O_3 were investigated by UV-vis analysis, which is illustrated in **Fig. S4**a. The UV-vis spectrum is a characteristic of the absorption of radiant energy of visible light and the transition of the outer electrons of the atoms from lower to higher energy level.[1] In this work, the UV absorption study is performed in the range of 290 to 800 nm at room temperature. The resulted spectrum contains a wider and intense peak at 307 nm (an enlarged view is shown in the inset of **Fig. S4a** and this is the characteristic band of synthesized Co_3O_4/Al_2O_3 NSs. The obtained UV absorption band is authenticated for the transition of valance electron of prepared NSs from lower to higher energy level.[2-4] According to the equation (6); the calculated band gape energy is 4.04 eV.

$$E_{bg} = 1240/\lambda_{max} \qquad (6)$$

Here, E_{bg} = band-gap energy, λ_{max} = maximum absorbed wave length.[5,6]

To identify the functional groups existing in the prepared NSs, the Co_3O_4/Al_2O_3 NSs were investigated by Fourier-transform infrared (FTIR) spectroscopy, which is presented in **Fig. S4**b. The FTIR is performed at range of 450–4000 cm^{-1} and resultant peaks are obtained from the corresponding atomic or molecular vibration. As it is seen in FTIR spectrum, the several major peaks at 536, 658, 1634 and 3414 cm^{-1} are observed. The two major peaks at 536 and 658 cm^{-1} are associated to the stretching vibrations of Co–O. This observation is consistent with those reported by previous authors.[7-9] The peaks at 1634 and 3400 cm^{-1} are assigned to O-H stretching vibration mode[10-13] due to the adsorption of water from environment.

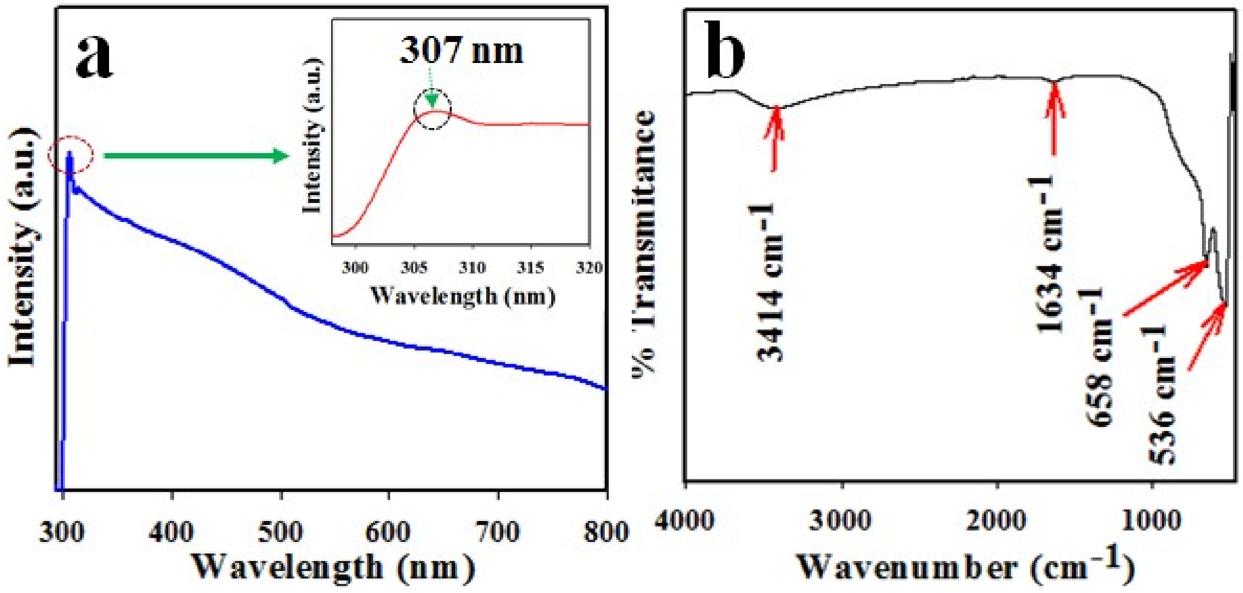

Fig. S4. Optical characterization of doped Co_3O_4/Al_2O_3 nanomaterials. (a) UV/vis. Spectroscopy and (b) FTIR spectroscopy

XPS analysis of doped Co_3O_4/Al_2O_3 nanomaterials:

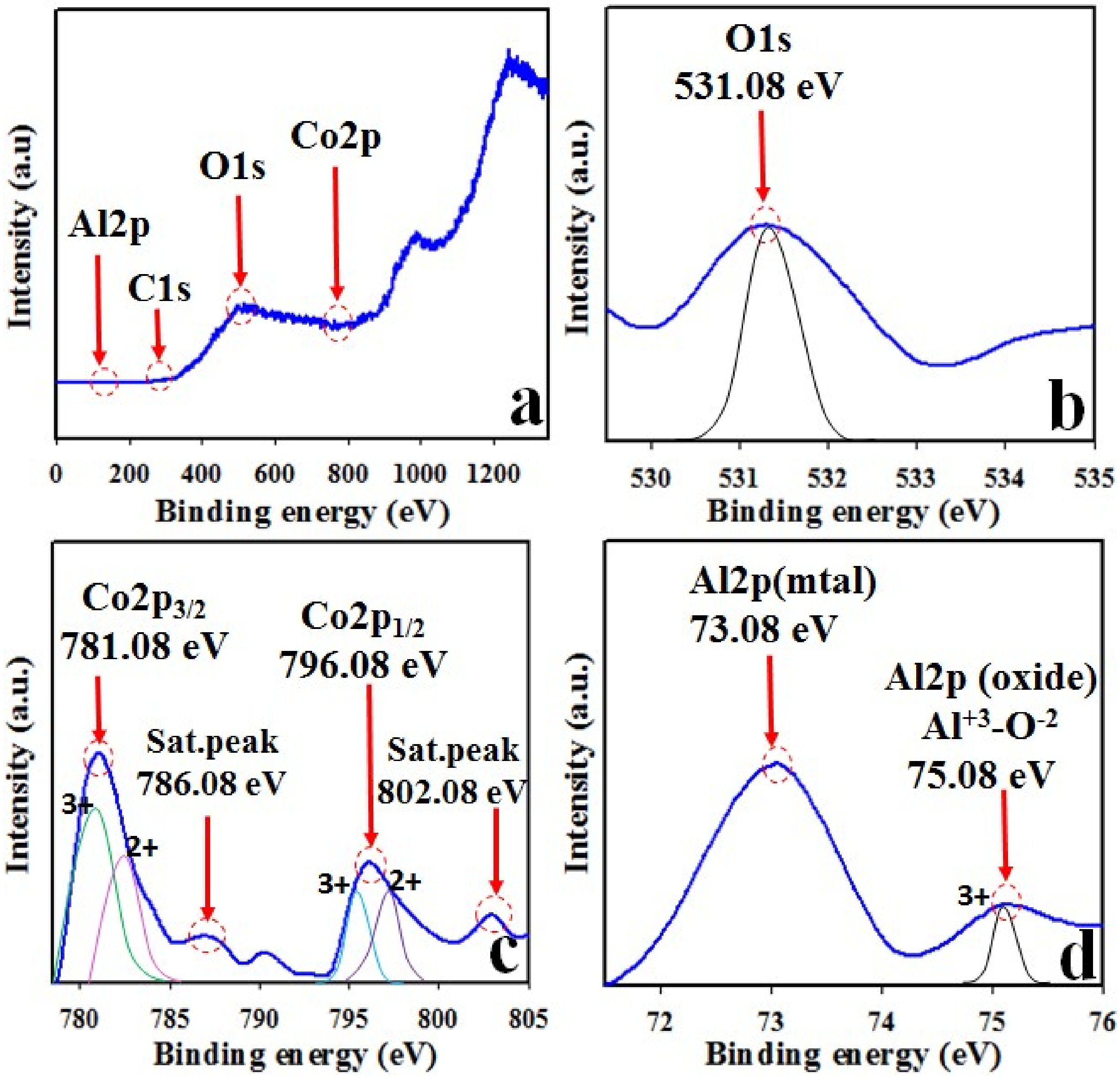

Fig. S5. XPS analysis of doped Co_3O_4/Al_2O_3 nanomaterials

References:

1. M. M. Rahman, A. Jamal, S. B. Khan and M. Faisal, J. Phys. Chem. C, 2011, 115, 9503–9510.
2. M. M. Rahman, G. Gruner, M. S. Al-Ghamdi, M. A. Daous, S. B. Khan and A. M. Asiri, Chemistry Central Journal, 2013, 7, 60.
3. R. Ramasamy, K. Ramachandran, G. G. Philip, R. Ramachandran, H. A. Therese and G. G. kumar, RSC Adv., 2015, 5, 76538-76547.
4. S. Kumar and A.K. Ojha, RSC Adv., 2016, 6, 8651–8660.
5. M. M. Rahman, M. M. Alam, A. M. Asiri and M. A. Islam, Talanta, 2017, 170, 215–223.
6. M. M. Rahman, M. M. Alam, A. M. Asiri and M. A. Islam, RSC Adv., 2017, 7, 22627–22639.
7. S. Shahabuddin, N. M. Sarih, F. H. Ismail, M. M. Shahid and N. M. Huang, RSC Adv., 2015, 5, 83857-83867.
8. X. Xia, J. Tu, Y. Zhang, Y. Mai, X. Wang, C. Gu and X. Zhao, RSC Advances, 2012, 2, 1835–1841.

9. G. A. M. Ali, O. A. Fouad, S. A. Makhlouf, M. M. Yusoff and K. F. Chong, J. Solid State Electrochem, 2014, 18, 2505–2512.
10. H. Xu, Z. Hai, J. Diwu, Q. Zhang, L. Gao, D. Cui, J. Zang, J. Liu and C. Xue, Journal of Nanomaterials, 2015, 8.
11. X. Huang, Z. Chen, T. Gao, Q. Huang, F. Niu, L. Qin and Y. Huang, Energy Technol., 2013, 1, 751 – 756.
12. M. Nidhin, K. J. Sreeram and B. U. Nair, Chemical Engineering Journal, 2012, 185, 352– 357.
13. V. K. Patel, J. R. Saurav, K. Gangopadhyay, S. Gangopadhyay and S. Bhattachary, RSC Adv., 2015, 5, 21471–21479.
